# Efficient Estimation of Nucleotide Diversity and Divergence Using Callable Loci (and More)

**DOI:** 10.1093/molbev/msaf282

**Published:** 2025-11-22

**Authors:** Cade Mirchandani, Erik Enbody, Timothy B Sackton, Russ Corbett-Detig

**Affiliations:** Department of Biomolecular Engineering, University of California, Santa Cruz, Santa Cruz, CA 95064, USA; Genomics Institute, University of California, Santa Cruz, Santa Cruz, CA 95064, USA; Department of Biomolecular Engineering, University of California, Santa Cruz, Santa Cruz, CA 95064, USA; Department of Computational Biology, Cornell University, Ithaca, NY 14853, USA; Informatics Group, Harvard University, Cambridge, MA 02138, USA; Department of Biomolecular Engineering, University of California, Santa Cruz, Santa Cruz, CA 95064, USA; Genomics Institute, University of California, Santa Cruz, Santa Cruz, CA 95064, USA

**Keywords:** population genomics, nucleotide diversity, divergence estimation, Callable loci, large-scale genomic datasets

## Abstract

The increasing scale of population genomic datasets presents computational challenges in estimating summary statistics such as nucleotide diversity (*π*) and divergence (*d*_xy_). Accurate estimates of diversity require knowledge of missing data, and existing tools require all-site VCFs. However, generating these files is computationally expensive for large datasets. Here, we introduce Callable Loci And More (clam), a tool that leverages callable loci—determined from depth information—to estimate population genetic statistics using a variant-only VCF. This approach offers improvements in storage footprint and computational performance compared to contemporary methods. We validate clam's accuracy using simulated data, demonstrating that it produces estimates of *π*, *d*_xy_, and fixation index (*F*_ST_) identical to those from all-site VCF approaches. We then benchmark clam using a large muskox dataset and demonstrate that it produces accurate estimates of *π* while substantially reducing runtime requirements compared to current best-practice methods. clam provides an efficient and scalable alternative for population genomic analyses, facilitating the study of increasingly large and diverse datasets. clam is available as a standalone program and integrated into snpArcher for efficient reproducible population genomic analysis.

## Introduction

In recent years, rapid advancements in sequencing technology have dramatically lowered the cost of genome sequencing, enabling the generation of numerous, large population resequencing datasets (Weigel and Mott 2009; Shaffer et al. 2022; All of Us Research Program Genomics Investigators 2024). To analyze these large datasets effectively, there is a pressing need for tools that can efficiently handle the scale and complexity of high-throughput genomic data while maintaining analytical accuracy (Hendricks et al. 2018).

Nucleotide diversity (*π*), divergence (*d*_xy_), heterozygosity, and fixation index (*F*_ST_) are fundamental population genetic summary statistics that summarize key aspects of population history and structure. *π* measures genetic variation within populations (Nei and Li 1979), *d*_xy_ quantifies genetic distance between populations (Nei and Li 1979), heterozygosity reflects genetic diversity at the individual level, and *F*_ST_ measures population differentiation (Hudson et al. 1992; Bhatia et al. 2013). Accurate estimation of these statistics is essential for inferring demographic history, detecting selection, and understanding speciation processes (Hendricks et al. 2018; Luikart et al. 2018; Hohenlohe et al. 2021).

These fundamental population genetic parameters are commonly estimated using polymorphism data stored in variant call format (VCF) files (Danecek et al. 2011). Several population genomics tools and libraries can estimate these parameters from VCFs, including vcftools (Danecek et al. 2011), pixy (Korunes and Samuk 2021), PopGenome (Pfeifer et al. 2014), and scikit-allel (Miles et al. 2024). However, standard VCF files (from here on referred to as “variant-only VCFs”) pose challenges for estimating statistics that rely on per-base comparisons such as *π* and *d*_xy_. Variant-only VCFs do not record genotypes at invariant sites, requiring tools to decide how to treat these sites. While some programs, such as scikit-allel, allow a user-specified accessibility mask to determine which sites should be treated as truly invariant, many tools simply assume that these sites are homozygous for the reference allele, leading to systematic underestimation of diversity statistics (Korunes and Samuk 2021). Importantly, these estimators themselves are not statistically biased; rather, the apparent bias arises from mismeasurement of the denominator in per-base-pair calculations, where incorrect assumptions about unobserved sites result in erroneous counts of the total number of base pairs that were actually assayed.

To address this issue, an “all-site” VCF that includes both variant and invariant positions can be used as input to calculate these statistics (Korunes and Samuk 2021). This solution produces more accurate estimates of diversity by explicitly recording which sites were successfully genotyped across all samples, allowing proper enumeration of the denominator in per-base calculations. However, the creation, storage, and analysis of all-site VCFs pose computational challenges as genomic datasets grow larger, making them impractical to produce, analyze, and store. Additionally, filtering approaches for all-site VCFs often differ from those applied to variant-only VCFs, introducing potentially unaccounted-for confounding effects to population genomic analyses. Probabilistic methods, such as ANGSD (Korneliussen et al. 2014), offer an alternative approach. ANGSD uses genotype likelihoods across all sites to derive allele frequencies, and several nucleotide diversity parameters can be subsequently estimated using custom scripts. However, this approach differs from methods that use hard-called genotypes, which offer advantages when sequencing depth allows for confident genotype calls that simplify parameter estimation (ie based on observed genotypes). Thus, there remains a need for tools that accurately estimate diversity from genotype calls.

One such VCF-based solution is to leverage a variant-only VCF and sequencing depth to determine whether each site in the genome is sufficiently covered to be considered “callable” for each sample. This information can be used to fill in the gaps of a variant-only VCF by identifying regions where sequencing coverage was sufficient to call genotypes, but are not present in the VCF, allowing these positions and their corresponding callable genotypes to be counted as invariant in diversity calculations. However, there are no maintained tools that both efficiently generate per-site callable sample counts and use this information to estimate population genetic statistics. Here, we present Callable Loci And More (clam), a command-line tool that performs 2 key functions: (i) generating per-site callable sample counts from sequencing depth (hereafter “callable loci”) and (ii) using these counts with a VCF to estimate population genetic statistics. We show that clam produces accurate estimates of *π*, *d*_xy_, and *F*_ST_ by correctly accounting for the denominator in per-base calculations, while requiring less disk space and less compute time than all-site approaches, enabling population genetic insights from increasingly vast datasets.

## Methods

### Implementation

clam is a command-line tool written in Rust with 2 primary functions: generating per-site callable sample counts from depth information and calculating population genetic statistics using these counts alongside VCF data. These functions are implemented as 2 independent subcommands: clam loci and clam stat.

The loci subcommand determines how many samples are callable at each genomic position by analyzing per-sample sequencing depth from either D4 files (Hou et al. 2021) or genomic variant call format files (GVCFs). D4 files are highly efficient compressed estimates of sample depth for every site and are easily produced (eg using mosdepth) from alignment (ie bam) files. Based on user-specified depth thresholds, clam loci identifies which samples have sufficient coverage for reliable genotype calling at each site and outputs these per-site callable sample counts. When population labels are provided, it generates per-population counts, enabling accurate between-population comparisons. Output formats include D4 (default) or BED files.

The *stat* subcommand accepts a VCF and the callable loci file as inputs to calculate *π* and *d*_xy_, *F*_ST_ (if applicable population labels are provided), and per-sample heterozygosity in genomic windows. All statistics are calculated simultaneously in a single pass through the input data. For each site in the VCF, clam counts pairwise differences and comparisons between samples, excluding missing genotypes. At sites not present in the VCF, the callable loci file provides the number of callable samples, enabling correct calculation of pairwise comparisons. This approach ensures accurate per-base diversity estimates by using the appropriate denominator at both variant and invariant sites.

Source code and documentation for *clam* are available at https://github.com/cademirch/clam and can be installed via Bioconda (Grüning et al. 2018) or by compiling from source.

### Simulated Dataset Generation

To validate the accuracy of clam's callable loci approach for estimating diversity statistics, we developed a Snakemake (Mölder et al. 2021) workflow to simulate realistic sequencing datasets from simulated genealogies with known population genetic parameters.

We simulated genetic variation in a 100 kb region of chromosome 2L from the *Drosophila melanogaster* genome (GCF_000001215.4) using msprime (Baumdicker et al. 2022) and stdpopsim (Adrion et al. 2020), with Ne = 1,720,600, mu = 5.49e-09, and recombination rate = 2.40463e-08. We simulated 20 individuals and randomly assigned them to 2 populations for between-population comparisons. This simulation produced a “perfect” VCF containing all true variants, which we used to generate individual-specific sequencing reads. We simulated 150 bp paired-end Illumina reads using Mason (Holtgrewe 2010), with per-sample coverage drawn from a gamma distribution (shape *k* = 4, mean = 10×) to mimic the sample quality variation commonly observed in population genomic studies. We aligned the simulated reads with BWA MEM (Li 2013) and generated D4 depth profiles using mosdepth (Pedersen and Quinlan 2018).

We generated variant calls using genome analysis toolkit (GATK) (McKenna et al. 2010) HaplotypeCaller, GenomicsDBImport, and GenotypeGVCFs. We used HaplotypeCaller to generate per-sample GVCFs with --emit-ref-confidence GVCF, capturing per-base depth and genotype quality information across all sites. We combined the GVCFs using GenomicsDBImport and then performed joint genotyping using GenotypeGVCFs, generating both variant-only VCFs (default) and all-site VCFs (--all-site flag) for comparison. We filtered VCFs by excluding indels, retaining only biallelic SNPs, and setting genotypes to missing when depth <2 or reference genotype quality (RGQ) < 10. For invariant sites in all-site VCFs, we applied the same thresholds to maintain consistency. We generated callable loci using clam loci directly on the per-sample GVCFs with matching thresholds (depth ≥2 and genotype quality [GQ] ≥10).

### Empirical Dataset

To benchmark the computational performance of clam at scale, we reanalyzed a population genomic dataset muskox (*Ovibos moschatus*) (Pečnerová et al. 2024). We selected this dataset as the muskox genome is relatively large (2.6 Gb) and low heterozygosity (0.6 × 10^−4^ to 2.4 × 10^−4^), both features that will produce very large all-site VCFs. We obtained sequencing reads for 59 samples and generated per-sample GVCFs using snpArcher (Mirchandani et al. 2024). Briefly, we aligned reads to the muskox reference genome (Lok et al. 2024) using BWA MEM. We removed PCR duplicates from the alignments using Sentieon *driver* (Kendig et al. 2019) and then followed the same joint variant calling and callable loci generation approach described for the simulated datasets, with the following modifications to the filtering thresholds: depth of ≥10, genotype quality ≥10, as well as applying GATK hard filters to variant sites.

### Comparison to Existing Methods

We generated 100 simulated datasets, and for each, we estimated *π*, *d*_xy_, and *F*_ST_ using 3 approaches: (i) pixy with all-site VCFs as our accuracy baseline, representing current best practice for unbiased estimation with missing data; (ii) clam with all-site VCFs to verify algorithmic correctness; and (iii) clam with variant-only VCFs plus callable loci to validate our novel approach. This allowed us to confirm both that clam's algorithm correctly handles missing data and that the callable loci approach achieves equivalent accuracy without requiring all-site VCFs. For the real muskox dataset, we estimated *π*, *d*_xy_, and *F*_ST_ in 100 kb windows using both clam approaches (all-site VCFs and variant-only VCFs plus callable loci) and compared the results. We also compared runtime and storage requirements between the 2 approaches.

Full details and scripts for all analyses are available at https://github.com/cademirch/clam-manuscript.

## Results and Discussion

### clam Accurately Estimates Diversity Without All-Site VCFs

**Fig. 1.**
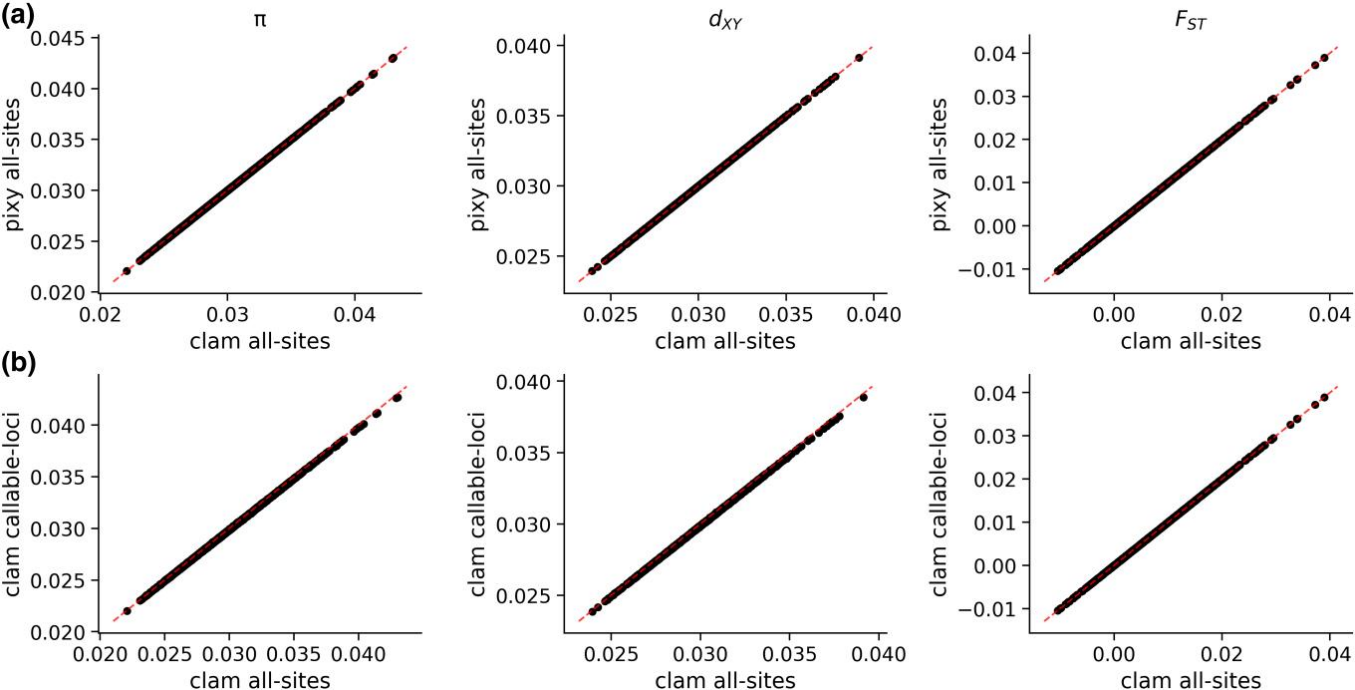
Comparison of diversity estimates between methods using simulated data. a) Estimates of *π*, *d*_xy_, and *F*_ST_ from clam versus pixy using identical all-site VCFs. b) Estimates from clam using all-site VCFs versus callable loci approach. Red dashed lines indicate *y* = *x*.

To validate clam's accuracy, we compared diversity estimates across methods using our simulated datasets. Our simulations generated VCFs with realistic patterns of missing data, with 10% to 15% of genotypes missing after quality filtering (Fig. S1), providing an appropriate test for clam's handling of incomplete data. First, we verified clam's algorithmic accuracy by comparing its estimates against pixy, which represents the current best practice for unbiased diversity estimation from VCFs with missing data. When provided with identical all-site VCFs, both tools produced identical estimates of *π*, *d*_xy_, and *F*_ST_ (*R*² = 1.00 for all statistics, Fig. 1a), demonstrating that clam correctly excludes missing genotypes and sites from its calculations.

Next, we tested whether the callable loci approach implemented in clam could produce accurate diversity estimates without an all-site VCF. We compared the estimates of *π*, *d*_xy_, and *F*_ST_ produced by clam using variant-only VCFs plus callable loci against those produced by clam using only all-site VCFs. To ensure comparable results, we used the same depth and quality thresholds when filtering the all-site VCF and generating callable loci from GVCFs. However, during joint genotyping, GenotypeGVCFs may reassign these values, potentially creating discrepancies between GVCF-based callable loci and the final filtered all-site VCF. Nonetheless, the callable loci approach in clam produced nearly identical results to the all-site approach (*R*² > 0.99 for *π* and *d*_xy_, *R*² = 1.00 for *F*_ST_; Fig. 1b) and minimal mean differences for *π* and *d*_xy_ (both ∼0.0001; Table S1). For *π* and *d*_xy_, the slight differences stem entirely from differences in the denominator (number of pairwise comparisons), while the numerator (pairwise differences) remains unchanged (Table S2). The discrepancies arise from GATK reassigning quality values during joint genotyping, causing slight differences in which samples are deemed callable at each site. To further validate the callable loci approach and demonstrate its flexibility, we repeated the above analysis using VCFs generated by bcftools and callable loci calculated from per-sample depth profiles (see Methods S1). The variant-only plus callable loci approach produced identical estimates to the all-site VCFs for *π*, *d*_xy_, and *F*_ST_ (*R*² = 1.00 for all statistics; Table S1).

Finally, we note that both pixy and clam underestimated diversity relative to the true values from our simulations (Table S3), reflecting the well-known reference bias effect, whereby non-reference alleles are systematically lost during read alignment (Brandt et al. 2015; Günther and Nettelblad 2019; Thorburn et al. 2023; Akopyan et al. 2025). While clam and the callable loci approach cannot overcome this inherent limitation of linear reference alignment, future integration with graph-based methods (Garrison et al. 2018) could help mitigate reference bias.

Beyond algorithmic accuracy, the quality of diversity estimates depends on appropriate filtering decisions. Selecting appropriate filtering thresholds is a complex topic that can substantially impact downstream analyses (Han et al. 2014; Pfeifer 2021). Thus, we recommend that users carefully select depth and quality thresholds based on their specific dataset characteristics and apply them consistently across both variant calling and callable loci generation. Additionally, users working with repetitive genomes may benefit from incorporating mappability masks (eg from RepeatMasker (Tarailo-Graovac and Chen 2009) or GenMAP (Pockrandt et al. 2020)) to exclude regions where high depth may not reflect reliable mapping.

### Empirical Validation and Computational Efficiency

Finally, we applied clam to an empirical dataset: resequencing of 59 muskox (Pečnerová et al. 2024), to further validate the callable loci approach on real data and quantify the computational burden associated with all-site VCF workflows. As with our simulations, we generated both all-site and variant-only VCFs, comparing diversity estimates from clam using the variant-only and callable loci against the estimates from the all-site VCF. Consistent with our simulation results, estimates generated by clam using the callable loci approach were highly similar to those estimates using the all-site VCF approach (*R*² = 0.99 for *π* and *d*_xy_, *R*² = 1.00 for *F*_ST_; Table S1). However, the computational costs of these 2 approaches differed substantially.

To compare computational requirements, we summed the runtime for all workflow steps from joint genotyping to diversity estimation (preceding steps were identical between approaches). Benchmarking was performed on a high-performance computing cluster with nodes containing 4 AMD EPYC 9684X processors (384 cores) and 2.3 TB RAM. While absolute runtimes will depend on computational infrastructure, the relative differences between the all-site and callable loci approaches provide a reliable metric of computational cost. In total, the all-site workflow required 468.85 h to complete, 1.94× greater than the 241.97 h required for the callable loci workflow (Table 1). This runtime difference stems from fundamental differences in data representation. All-site VCFs encode every genomic position as individual records, while the callable loci approach collapses invariant regions into genomic intervals. This advantage scales with dataset size; all-site VCFs grow linearly with sample count and genome size, whereas callable loci files remain compact regardless of sample number. As each workflow step must parse every VCF record, these I/O costs compound throughout the pipeline, making the callable loci approach particularly valuable for large-scale population genomic studies.

**Table 1.** Computational runtime requirements of all-site and variant-only workflows

Processing step	Approach		Increase	% of total difference
	Variant-only	All-site		
Generate callable loci	2.29	…	…	−1.01%
Joint genotyping	232.87	414.95	1.78×	80.25%
Filter invariant sites	…	29.96	…	13.21%
Filter variant sites	6.74	23.28	3.45×	7.29%
Estimate pi	0.07	0.66	9.43×	0.26%
**Total time**	241.97	468.85	1.94×	100.00%

Each value represents the sum of wall clock time (in hours) for all jobs within the workflow step. The “filtering variant sites” in the all-site workflow includes time to separate variant and invariant sites.

## Conclusion

clam provides a computationally efficient and scalable approach for estimating population genetic statistics from large genomic datasets. Its modular architecture enables flexible integration into diverse workflows: clam loci generates per-site callable sample counts from depth information or GVCFs, which clam stat uses to calculate accurate diversity estimates or can be exported for use with other tools. This design eliminates the need for computationally expensive all-site VCFs while still correctly accounting for the denominator in per-base calculations. We demonstrate that clam produces identical estimates to current best-practice methods when using all-site VCFs and that the callable loci approach yields nearly identical diversity and divergence estimates without requiring all-site VCFs. Furthermore, benchmarking on a large population genomics dataset shows that the callable loci approach reduces runtime nearly 2-fold compared to traditional all-site workflows. These advantages make clam particularly well-suited for studies involving massive sequencing datasets, notably those with larger genomes and low genetic diversity, where computational efficiency is critical. As genomic datasets continue to grow, clam offers an essential tool for researchers seeking accurate and efficient population genetic analyses.

## Supplementary Material

msaf282_Supplementary_Data

## Data Availability

Source code and documentation for clam are available at https://github.com/cademirch/clam. Raw sequence reads for the muskox dataset are available in the European Nucleotide Archive under study accession ID: PRJEB64293, and the reference genome is available under the NCBI GenBank assembly accession ID: GCA_041156055.1.

## References

[msaf282-B1] Adrion JR et al A community-maintained standard library of population genetic models. Elife. 2020:9:e54967. 10.7554/eLife.54967. 32573438 PMC7438115

[msaf282-B2] Akopyan M, Genchev M, Armstrong EE, Mooney JA. Reference genome choice compromises population genetic analyses. Cell. 2025. 10.1016/j.cell.2025.08.034. 40987293

[msaf282-B3] All of Us Research Program Genomics Investigators . Genomic data in the all of us research program. Nature. 2024:627:340–346. 10.1038/s41586-023-06957-x. 38374255 PMC10937371

[msaf282-B4] Baumdicker F et al Efficient ancestry and mutation simulation with msprime 1.0. Genetics. 2022:220:iyab229. 10.1093/genetics/iyab229. 34897427 PMC9176297

[msaf282-B5] Bhatia G, Patterson N, Sankararaman S, Price AL. Estimating and interpreting FST: the impact of rare variants. Genome Res. 2013:23:1514–1521. 10.1101/gr.154831.113. 23861382 PMC3759727

[msaf282-B6] Brandt DYC et al Mapping bias overestimates reference allele frequencies at the HLA genes in the 1000 Genomes Project phase I data. G3 (Bethesda). 2015:5:931–941. 10.1534/g3.114.015784. 25787242 PMC4426377

[msaf282-B7] Danecek P et al The variant call format and VCFtools. Bioinformatics. 2011:27:2156–2158. 10.1093/bioinformatics/btr330. 21653522 PMC3137218

[msaf282-B8] Garrison E et al Variation graph toolkit improves read mapping by representing genetic variation in the reference. Nat Biotechnol. 2018:36:875–879. 10.1038/nbt.4227. 30125266 PMC6126949

[msaf282-B9] Grüning B et al Bioconda: sustainable and comprehensive software distribution for the life sciences. Nat Methods. 2018:15:475–476. 10.1038/s41592-018-0046-7. 29967506 PMC11070151

[msaf282-B10] Günther T, Nettelblad C. The presence and impact of reference bias on population genomic studies of prehistoric human populations. PLoS Genet. 2019:15:e1008302. 10.1371/journal.pgen.1008302. 31348818 PMC6685638

[msaf282-B11] Han E, Sinsheimer JS, Novembre J. Characterizing bias in population genetic inferences from low-coverage sequencing data. Mol Biol Evol. 2014:31:723–735. 10.1093/molbev/mst229. 24288159 PMC3935184

[msaf282-B12] Hendricks S et al Recent advances in conservation and population genomics data analysis. Evol Appl. 2018:11:1197–1211. 10.1111/eva.12659.

[msaf282-B13] Hohenlohe PA, Funk WC, Rajora OP. Population genomics for wildlife conservation and management. Mol Ecol. 2021:30:62–82. 10.1111/mec.15720. 33145846 PMC7894518

[msaf282-B14] Holtgrewe M. 2010. Mason—A read simulator for second generation sequencing data. *Technical Report FU Berlin* [Internet]. Available from: http://publications.imp.fu-berlin.de/962/

[msaf282-B15] Hou H, Pedersen B, Quinlan A. Balancing efficient analysis and storage of quantitative genomics data with the D4 format and d4tools. Nat Comput Sci. 2021:1:441–447. 10.1038/s43588-021-00085-0. 35936573 PMC9355464

[msaf282-B16] Hudson RR, Slatkin M, Maddison WP. Estimation of levels of gene flow from DNA sequence data. Genetics. 1992:132:583–589. 10.1093/genetics/132.2.583. 1427045 PMC1205159

[msaf282-B17] Kendig KI et al Sentieon DNASeq variant calling workflow demonstrates strong computational performance and accuracy. Front Genet. 2019:10:736. 10.3389/fgene.2019.00736. 31481971 PMC6710408

[msaf282-B18] Korneliussen TS, Albrechtsen A, Nielsen R. ANGSD: analysis of next generation sequencing data. BMC Bioinformatics. 2014:15:356. 10.1186/s12859-014-0356-4. 25420514 PMC4248462

[msaf282-B19] Korunes KL, Samuk K. pixy: unbiased estimation of nucleotide diversity and divergence in the presence of missing data. Mol Ecol Resour. 2021:21:1359–1368. 10.1111/1755-0998.13326. 33453139 PMC8044049

[msaf282-B20] Li H. 2013. Aligning sequence reads, clone sequences and assembly contigs with BWA-MEM [preprint]. arXiv, arXiv:1303.3997.

[msaf282-B21] Lok S et al Chromosomal-level reference genome assembly of muskox (*Ovibos moschatus*) from Banks Island in the Canadian Arctic, a resource for conservation genomics. Sci Rep. 2024:14:21023. 10.1038/s41598-024-67270-9. 39284808 PMC11405533

[msaf282-B22] Luikart G et al Population genomics: advancing understanding of nature. In: Population genomics. Cham: Springer International Publishing; 2018. p. 3–79.

[msaf282-B23] McKenna A et al The genome analysis toolkit: a MapReduce framework for analyzing next-generation DNA sequencing data. Genome Res. 2010:20:1297–1303. 10.1101/gr.107524.110. 20644199 PMC2928508

[msaf282-B24] Miles A et al cggh/scikit-allel: v1.3.13. [Computer software]. Zenodo; 2024, September 17. https://zenodo.org/records/13772087.

[msaf282-B25] Mirchandani CD et al A fast, reproducible, high-throughput variant calling workflow for population genomics. Mol Biol Evol. 2024:41:msad270. 10.1093/molbev/msad270. 38069903 PMC10764099

[msaf282-B26] Mölder F et al Sustainable data analysis with Snakemake. F1000Res. 2021:10:33. 10.12688/f1000research.29032.2. 34035898 PMC8114187

[msaf282-B27] Nei M, Li WH. Mathematical model for studying genetic variation in terms of restriction endonucleases. Proc Natl Acad Sci U S A. 1979:76:5269–5273. 10.1073/pnas.76.10.5269. 291943 PMC413122

[msaf282-B28] Pečnerová P et al Population genomics of the muskox’ resilience in the near absence of genetic variation. Mol Ecol. 2024:33:e17205. 10.1111/mec.17205. 37971141

[msaf282-B29] Pedersen BS, Quinlan AR. Mosdepth: quick coverage calculation for genomes and exomes. Bioinformatics. 2018:34:867–868. 10.1093/bioinformatics/btx699. 29096012 PMC6030888

[msaf282-B30] Pfeifer B, Wittelsbürger U, Ramos-Onsins SE, Lercher MJ. PopGenome: an efficient Swiss army knife for population genomic analyses in R. Mol Biol Evol. 2014:31:1929–1936. 10.1093/molbev/msu136. 24739305 PMC4069620

[msaf282-B31] Pfeifer SP . Studying mutation rate evolution in primates-the effects of computational pipelines and parameter choices. Gigascience. 2021:10:giab069. 10.1093/gigascience/giab069. 34673929 PMC8529961

[msaf282-B32] Pockrandt C, Alzamel M, Iliopoulos CS, Reinert K. GenMap: ultra-fast computation of genome mappability. Bioinformatics. 2020:36:3687–3692. 10.1093/bioinformatics/btaa222. 32246826 PMC7320602

[msaf282-B33] Shaffer HB et al Landscape genomics to enable conservation actions: The California Conservation Genomics Project. J Hered. 2022:113:577–588. 10.1093/jhered/esac020. 35395669

[msaf282-B34] Tarailo-Graovac M, Chen N. Using RepeatMasker to identify repetitive elements in genomic sequences. Curr Protoc Bioinformatics. 2009:4:4.10.1–4.10.14. 10.1002/0471250953.bi0410s25. 19274634

[msaf282-B35] Thorburn D-MJ et al Origin matters: using a local reference genome improves measures in population genomics. Mol Ecol Resour. 2023:23:1706–1723. 10.1111/1755-0998.13838. 37489282

[msaf282-B36] Weigel D, Mott R. The 1001 genomes project for Arabidopsis thaliana. Genome Biol. 2009:10:107. 10.1186/gb-2009-10-5-107. 19519932 PMC2718507

