# Correction to: Efficient Estimation of Nucleotide Diversity and Divergence Using Callable Loci (and More)

**DOI:** 10.1093/molbev/msag192

**Published:** 2026-08-10

This is a correction to: Cade Mirchandani, Erik Enbody, Timothy B Sackton, Russ Corbett-Detig, Efficient Estimation of Nucleotide Diversity and Divergence Using Callable Loci (and More), *Molecular Biology and Evolution*, Volume 42, Issue 12, December 2025, msaf282, https://doi.org/10.1093/molbev/msaf282

In the originally published version of this manuscript, a Conflict of Interest statement was omitted.

The following has been added to a newly created “Conflicts of Interest” section:

“R.C.-D. reports consulting for International Responder Systems. This consulting relationship had no role in the design, conduct, interpretation, or publication of this study. The authors declare no other conflicts of interest”.

