# Supplementary material for: Efficient Estimation of Nucleotide Diversity and Divergence Using Callable Loci (and More)
Source: Mol Biol Evol. 2025 Nov 22;42(12):msaf282. doi: 10.1093/molbev/msaf282 (PMC12697346; doi:10.1093/molbev/msaf282)
Supplement: msaf282_Supplementary_Data [file msaf282_supplementary_data.zip › supplementary_information.pdf]

Supplementary Information

Table S1. Comparison of diversity statistics calculated using different approaches. R^2 values, mean differences, and relative differences for nucleotide diversity (π), absolute divergence (dxy), and fixation index (FST) comparing: (1) clam versus pixy using all-sites VCFs, and (2) clam using all-sites VCFs versus clam using variants-only VCFs with callable loci. Results shown for simulated data processed with both GATK and bcftools pipelines, and empirical muskox data. [table_s1.xlsx]

Table S2. Decomposition of differences between all-sites and callable loci approaches for simulated data processed with GATK. Mean differences and relative differences in the numerator (number of pairwise differences) and denominator (number of pairwise comparisons) components of nucleotide diversity (π) and absolute divergence (dxy) calculations. [table_s2.xlsx]

Table S3. Comparison of nucleotide diversity (π) and absolute divergence (dxy) estimates from clam and pixy against known true values from simulations. [table_s3.xlsx]

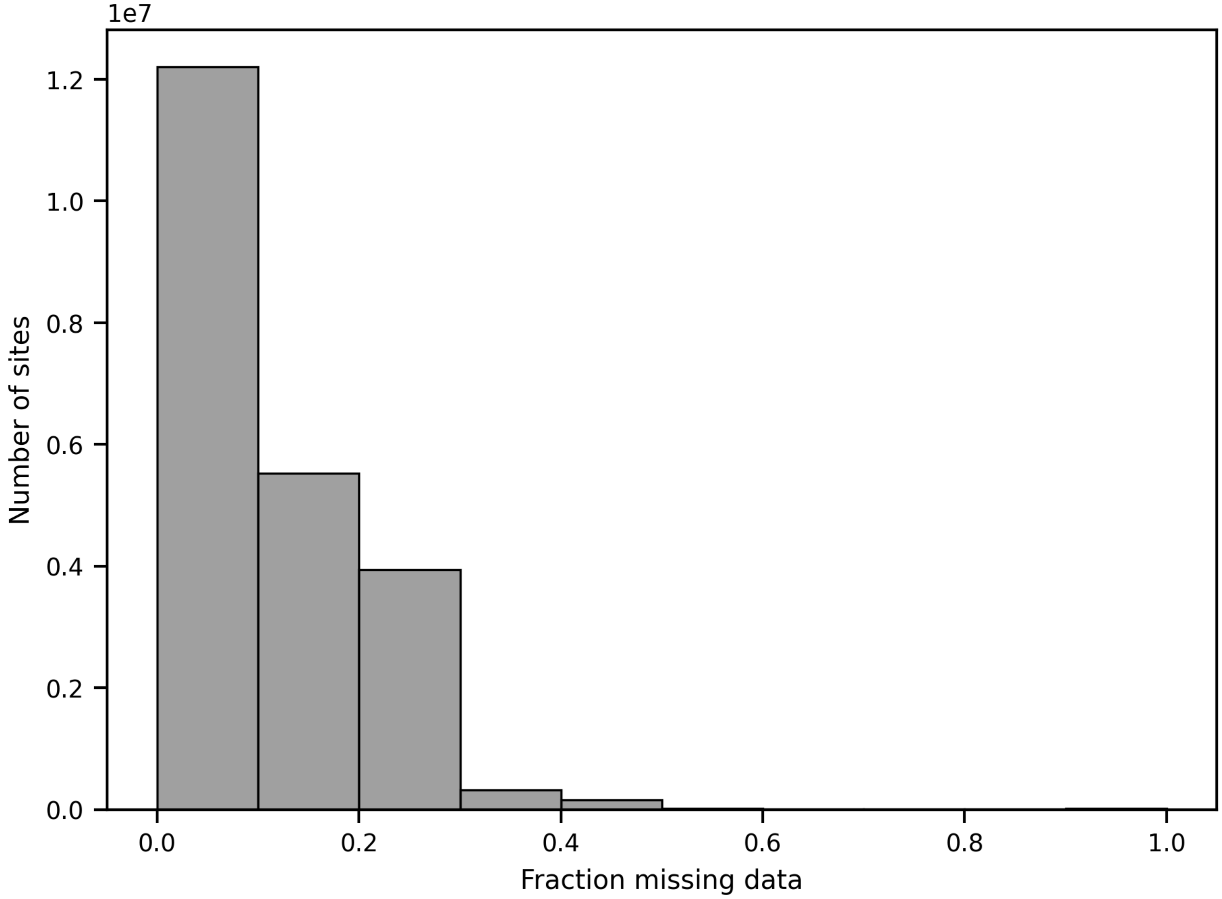

Figure S1. Distribution of missing data in simulated VCFs. [figure_s1.png]

Methods

bcftools variant calling and callable loci generation

We used bcftools mpileup with -q 30 to exclude reads with mapping quality <30, generating a multi-sample pileup from the aligned BAM files. We then used bcftools call with the multiallelic caller (-m flag) to generate all-sites VCFs and with -mv to generate variants-only VCFs. We filtered bcftools VCFs identically to GATK for variant types (biallelic SNPs only) and set genotypes to missing when depth <2. To generate the callable loci file, we first made per sample depth files (D4) by using mosdepth with the flag -Q 30 to match the mapping quality filter using the mpileup. We then used clam loci with these D4 files, applying a depth ≥2 threshold to match our VCF filtering.
