# Supplementary figures and images for: Efficient Estimation of Nucleotide Diversity and Divergence Using Callable Loci (and More)
Source: Mol Biol Evol. 2025 Nov 22;42(12):msaf282. doi: 10.1093/molbev/msaf282 (PMC12697346; doi:10.1093/molbev/msaf282)

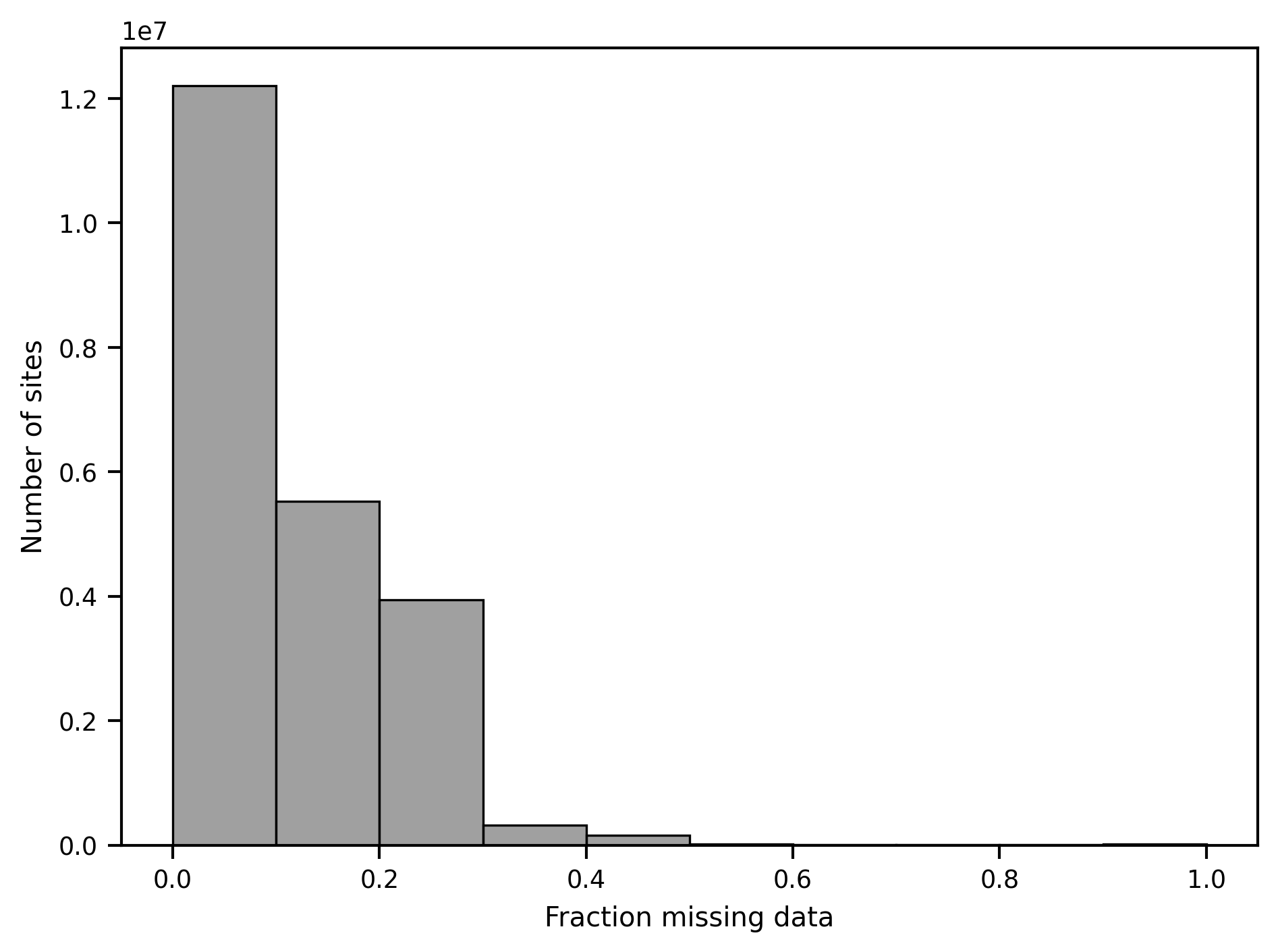

Supplement: msaf282_Supplementary_Data [file msaf282_supplementary_data.zip › figure_s1.png]
